# Evaluation of 147 Kampo prescriptions as novel protein tyrosine phosphatase 1B (PTP1B) inhibitory agents

**DOI:** 10.1186/1472-6882-14-64

**Published:** 2014-02-20

**Authors:** Toshihisa Onoda, Wei Li, Koji Higai, Kazuo Koike

**Affiliations:** 1 Faculty of Pharmaceutical Sciences, Toho University, Miyama 2-2-1, Funabashi, Chiba 274-8510, Japan; 2 Toho University Sakura Medical Center, Shimoshidu 564-1, Sakura, Chiba 285-8741, Japan

**Keywords:** Kampo, Protein tyrosine phosphatase 1B, Insulin-resistance Type 2 diabetes mellitus, *Rhei Rhizoma*

## Abstract

**Background:**

Protein tyrosine phosphatase (PTP) 1B, a negative regulator of the insulin and leptin signaling pathways, is currently considered a promising target for the development of novel therapeutic approaches used to treat insulin-resistant type 2 diabetes mellitus (IR-T2DM). In this study, we examined the PTP1B inhibitory activity of 147 Japanese prescription Kampo formulations to evaluate their potential for clinical application in IR-T2DM treatment.

**Methods:**

We specifically defined the prescribed daily dose as 1 Unit (U), and 147 Japanese prescription Kampo formulations were screened for PTP1B inhibitory activity at a final concentration of 0.1 mU/mL. We investigated the dependence of the inhibitory activity on the concentration of the Kampo formulations that exhibited high PTP1B inhibitory activity. Their inhibition mode by kinetic analysis, inhibitory selectivities against four homologous PTPs (TCPTP, VHR, SHP-1 and SHP-2) and cellular activity in the insulin-signaling pathway by increasing the insulin-stimulated Akt phosphorylation level in human hepatocellular liver carcinoma HepG2 cells, were also investigated. The statistical partial least squares regression method was used to identify the crude drugs with the greatest contribution to the PTP1B inhibitory activity of the Kampo formulations.

**Results:**

Daiokanzoto, Masiningan, Tokakujokito, Keimakakuhanto and Choijokito exhibited high PTP1B inhibitory activity, which was concentration-dependent. Daiokanzoto, Masiningan and Tokakujokito inhibited PTP1B by mixed inhibition modes and exhibited different inhibitory selectivities against four homologous PTPs. Masiningan also exhibited cellular activity. Statistical analyses indicated that the constituent crude drug *Rhei Rhizoma* provided the greatest contribution to the PTP1B inhibitory activity of these Kampo formulations.

**Conclusions:**

High PTP1B inhibitory activity was predominantly associated with formulations that were classified as Jyokito in Kampo medicine and with a modern clinical indication of constipation. Currently, there is no clinical treatment for IR-T2DM that uses a mechanism of action based on PTP1B inhibition. Thus, we propose the Kampo formulations identified in this study as strong PTP1B inhibitors, which could be developed as clinical therapeutic agents to treat IR-T2DM.

## Background

The incidence of type 2 diabetes mellitus (T2DM) is increasing worldwide [1]. A major subtype of T2DM is insulin-resistant T2DM (IR-T2DM), which mainly develops when insulin secretion in peripheral tissues is unable to compensate for insulin resistance [2]. IR-T2DM is of importance because it is associated with multiple complications such as cardiovascular anomalies [3]. Although the first-line treatment for IR-T2DM usually includes a healthy diet and exercise, patients with diabetes that cannot be controlled with healthy diet and exercise alone are treated with drugs, such as sulfonylureas (SU), dipeptidyl peptidase (DPP)-4 inhibitors, biguanides and thiazolidine derivatives [4,5]. However, because of the adverse effects and the presence of nonresponders, current treatments using these drugs have been limited. For example, pioglitazone, a thiazolidine derivative, which is currently used widely in monotherapy or combination therapy for IR-T2DM has serious adverse effects, including angioneurotic edema and cardiac failure [6,7]. The World Health Organization (WHO) has also recently reported bladder cancer as a new side effect [8]. Thus, there is a need to develop new approaches for the prevention and treatment of IR-T2DM. Tyrosine phosphatase (PTP) 1B, which is a negative regulator of insulin and leptin signaling pathways, is a promising target for the development of IR-T2DM treatment.

PTP1B dephosphorylates the insulin receptor and leptin receptor linked enzyme Janus kinase 2. It is an intracellular phosphatase that is localized on the cytoplasmic surface of the endoplasmic reticulum and is ubiquitously expressed in insulin target tissues, such as liver, muscle and adipose tissues [9]. PTP1B inhibition has been shown *in vivo* to increase insulin and leptin activity and results in normalized blood glucose levels and reduced adiposity [10,11]. In addition, PTP1B inhibition may protect against aging and has been studied from a broad perspective [12]. PTP1B inhibitors have gained much attention for their therapeutic value associated with their novel mode of action and are actively pursued in the development of new drugs. Although some PTP1B inhibitors are undergoing clinical trials, currently there are no PTP1B inhibitors available for clinical use.

A total of 148 prescription Kampo formulations consisting of 185 crude drugs are currently covered by Japan’s national health insurance. There are 147 types of oral formulations among 148 prescription Kampo formulations. These Kampo formulations are in clinical use for a variety of diseases, which are primarily based on traditional clinical theories. However, due to the elucidation of the mechanisms of action of Kampo formulations in recent years, evidence-based clinical applications are starting to be developed, as exemplified by Daikenchuto [13]. Kampo formulations consist of a combination of multiple crude drugs and are characterized by their exertion of a therapeutic effect as a multicomponent system, in which functional mechanisms of individual components are integrated. Thus, it is difficult to understand the cause-and-effect relationships. However, we consider these formulations to be beneficial for the treatment of metabolic disorders, such as IR-T2DM, which require multiple therapeutic effects. Kampo formulations, such as Goshajinkigan, can be used for the treatment of diabetes complications, as demonstrated by an *in vivo* study [14], and Bofutsushosan for the prevention and treatment of obesity [15].

In this study, we report the results of an examination of the PTP1B inhibitory activity of 147 Japanese prescription Kampo formulations to evaluate their potential for clinical application for the treatment of IR-T2DM.

## Methods

### Materials

This study examined 147 prescription Kampo formulations, which represent the entire panel of oral Kampo formulations covered by Japan’s national health insurance. These formulations were manufactured by Tsumura & Co. (Tokyo, Japan); Kotaro Pharmaceutical Co., Ltd. (Osaka, Japan); Ohsugi Pharmaceutical Co., Ltd. (Osaka, Japan); Kracie Holdings, Ltd. (Tokyo, Japan); Sanwa Shoyaku Co., Ltd. (Tochigi, Japan); Taikoseido Pharmaceutical Co., Ltd. (Hyogo, Japan); or Toyo-Kampo Pharmaceutical Co., Ltd. (Osaka, Japan). *Rhei Rhizoma* and *Cannabisi Fructus* were purchased from Tochimoto Tenkaido Co., Ltd. (Osaka, Japan). PTP1B (human, recombinant), T-cell protein tyrosine phosphatase (TCPTP) (human, recombinant) and Vaccinia H1-related phosphatase (VHR) (human, recombinant) were purchased from Enzo Life Sciences, Inc. (Lausen, Switzerland), and ursolic acid (purity: 98.5%), sodium orthovanadate (purity: 90%), citrate buffer solution (pH 6.0), *p*-nitrophenyl phosphate (*p*-NPP), bovine serum albumin, Src homology domain 2-Containing Protein Tyrosine Phosphatase 1 (SHP-1) and Src homology domain 2-Containing Protein Tyrosine Phosphatase 2 (SHP-2) were obtained from Sigma-Aldrich Co., LLC. (St Louis, MO, USA). Sodium chloride (NaCl), dithiothreitol (DTT), sodium hydroxide (NaOH), tris (hydroxymethyl) aminomethane, polyoxyethylene (23) lauryl ether and insulin (human, recombinant) were purchased from Wako Pure Chemical Industries, Ltd. (Osaka, Japan), and ethylenediaminetetraacetic acid (EDTA) was obtained from Dojindo Co., Ltd. (Kumamoto, Japan). Human hepatocellular carcinoma cell lines were purchased from pG2 Japanese Cancer Research Resources Bank/Health Science Research Resources Bank Co., Ltd. (Tokyo, Japan), and fetal bovine serum was purchased from SAFC Biosciences, Inc. (Lenexa, KS, USA). Dulbecco’s modified Eagle’s medium (DMEM) was obtained from Nissui Co., Ltd. (Tokyo, Japan). *p*-Akt1/2/3 (Ser 473) and Akt1/2/3 (H-136) antibodies were purchased from Santa Cruz biotechnology, Inc. (Dallas, TX, USA). Goat anti-rabbit IgG-heavy and light chain antibody was purchased from Bethyl Laboratories, Inc. (Montgomery, TX, USA). Hybond-P PVDF membrane and ECL-plus were purchased from GE Healthcare Life-Science Co., Ltd. (Tokyo, Japan). PVDF Blocking Reagent, Can Get Signal Solution-I and Can Get Signal Solution-II were purchased from Toyobo Co., Ltd. (Tokyo, Japan). Anti-rabbit IgG-h + l goat antibody was purchased from Bethyl laboratories Inc. (Montgomery, TX).

### Sample preparation of Kampo medicines

Each Kampo formulation was dissolved at 1/1,000 of the daily dose (1 Unit), as indicated on the package insert, in 1 mL of purified water, and extracted by sonication at room temperature for 15 min. The mixture was then centrifuged at 12,000 rpm for 15 min, and the supernatant or supernatant diluent (in purified water) was used as the sample for the PTP1B inhibitory activity assay.

### Sample preparation of crude drugs

The crude drug of *Rhei Rhizoma* or *Cannabisi Fructus* (60 g) was dissolved in distilled water (1,000 mL) and decocted until the volume was reduced by half. The extract solutions were freeze-dried to obtain samples of the crude drug.

### PTP1B and other PTPs inhibitory activity assay

The PTP1B inhibitory activity was measured using *p*-NPP as the substrate. Next, 40-μL solution of PTP1B or VHR was dissolved in a buffer solution consisting of 0.06 M citric acid (pH 6.0), 0.1 M NaCl, 1 mM EDTA and 1 mM DTT. For TCPTP, SHP-1 and SHP-2, assay buffer (PH 7.0) was prepared using 25 mM Tris/HCl, 50 mM NaCl, 2 mM ethylenediaminetetraacetic acid, 5 mM dithiothreitol, 0.01% Brij35 and 1 mg/mL bovine serum albumin; 50 μL of 2 mM *p*-NPP for PTP1B, TCPTP, VHR and SHP-2; and 16 mM for SHP-1; and 10 μL of the sample or the sample diluent were added to a 96-well plate (final volume, 100 μL). After incubation at 37°C for 30 min, the reaction was terminated by adding 20 μL of 10 M NaOH. The reaction mixture was shaken on a microplate mixer for 30 s, and the amount of *p*-nitrophenol produced was determined by measuring the absorbance at 405 nm. The nonenzymatic hydrolysis of 2 mM *p*-NPP was corrected by measuring the increase in absorbance at 405 nm, which was obtained in the absence of PTP1B. In this study, ursolic acid, a known PTP1B inhibitor, was used as the positive control and exhibited an IC_50_ (50% inhibition concentration) of 4.3 μM, which was consistent with a previous report [16].

### Inhibition rate and IC_50_

The inhibition rate of PTP1B activity of each sample was determined on the basis of the *p*-nitrophenol production measured as an increase in absorbance (*ΔA*) at 405 nm of the sample solution over the initial 30 min, using the following formula:

$$\text{Inhibition}\;\text{rate}\;\left(\%\right)=\frac{1‒\left(\Delta A_{\text{sample}}‒\Delta A_{\text{blank}}\right)}{\left(\Delta A_{\text{control}}\right)}\times 100$$

An inhibition rate of 90% or higher was considered to indicate complete inhibition. The dependence of the inhibitory activity on concentration was investigated in Kampo samples that exhibited a complete inhibition over a final concentration range from 0.1 mUnit/mL to 0.001 mUnit/mL. In addition, the crude drugs were investigated in the concentration range from 0.5 μg/mL to 100 μg/mL. The inhibition rate at each concentration was then used to calculate the IC_50_ using linear regression analysis.

### Kinetics analysis

To elucidate the inhibition mode, the inhibition kinetics of high PTP1B inhibitory Kampo samples (Daiokanzoto, Masiningan and Tokakujokito) were analyzed using the Lineweaver–Burk method with various substrate concentrations of *p*-NPP (2, 4, 8, 16 mM). The initial reaction velocities were measured in the presence (the concentration from 2 to 7 μUnit/mL) and absence of the inhibitor [17].

### Cytotoxicity assay

HepG2 cells was cultured in DMEM, supplemented with 10% heat-inactivated fetal bovine serum in a humidified atmosphere containing 5% CO_2_ at 37°C. The cells were incubated with the samples (Daiokanzoto, Masiningan, Tokakujokito, Keimakakuhanto and Choijokito) from 0 to 50 mU/mL for three days. The number of living cells was determined using the Premix WST-1 Cell Proliferation Assay System (TaKaRa, Shiga, Japan), according to the manufacturer’s instructions.

### Akt phosphorylation assay

HepG2 cells were cultured in DMEM, supplemented with 10% heat-inactivated fetal bovine serum, at 37°C in a humidified atmosphere containing 5% CO_2_. After pretreatment with each compound at 37°C for 60 min, HepG2 cells (500 μL, 5 × 105 cells/well) in a 48-well plate were stimulated with 50 nmol/L insulin for 5 min at 37°C. After washing with cold phosphate buffered saline, the cells were lysed using sonication. The lysate was centrifuged at 15,000 rpm for 15 min, and the supernatant was separated on 12% SDS-PAGE. After transfer to a Hybond-P PVDF membrane, the membrane was blocked with PVDF Blocking Reagent at 4°C overnight and was probed with 0.4 μg/mL anti-AKT1/2/3 rabbit polyclonal antibody or anti-phosphorylated AKT1/2/3 rabbit polyclonal antibody in Can Get Signal Solution-I for 1 h at room temperature, followed by further incubation with 0.025 μg/mL HRP-labeled anti-rabbit IgG-h + l goat antibody in Can Get Signal Solution-II for 1 h at room temperature. The membrane was then washed three times with phosphate buffered saline-T, incubated with ECL-plus for 5 min at room temperature and analyzed using a Typhoon 9410. Sodium orthovanadate was used as the positive control [18].

### Partial least squares regression analyses

The partial least squares (PLS) regression method [19] was used for statistical analysis of the contributions of individual constituent crude drugs to the PTP1B inhibitory activity of Kampo formulations. With each constituent crude drug of the Kampo formulations as the regressor variable (*X*_m_) and the PTP1B inhibitory activity of the Kampo formulations as the response variable (*Y*), we generated a regression formula to compare the contributions from individual constituent crude drugs in this study, as follows:

$$\begin{array}{ll} \;Y=a_{0} & +a_{1}X_{1}+a_{2}X_{2}+a_{3}X_{3}+a_{4}X_{4}+\cdots +a_{m}X_{m}\\ & +f\left(\text{constant}\right), \end{array}$$

where m is the number of factors, n is the number of samples, *Y* is the response variable, and *X* is the regressor variable factor. The regression coefficient is *a*, and its components are expressed as *a*_*j*_ (j = 1 2 3…m).

In the PLS regression model, regression was performed using the latent variable *t*_*k*_ as the explanatory variable by the following formula:

$$\begin{array}{l} Y=\bar{Y}\;+\sum_{K=1}^{A}t_{k}q_{k}+e=\bar{Y}\;+T\cdot q+e\\ X=\bar{X}\;+\sum_{K=1}^{A}t_{k}{p_{k}}^{T}+E=\bar{X}\;+T\cdot P^{T}+E, \end{array}$$

where *q*_*k*_ is the coefficient for the *k*th component in *Y*, *p*_*k*_ is the *k*th weight vector in *X*, *A* is the number of latent variables for PLS, *e* is the difference of *Y*, and *E* is the difference of *X*. Here, ^*T*^ represents a transposed matrix. The number of latent variables for PLS, *A*, was determined using the cross-validation method. Cross-validation was performed as described below. The *n − 1* set consisting of *n* data subtracted from 1 set was used to estimate the model parameters. The predictive value Ŷ for the response Y was obtained for the 1 set that was subtracted. The same procedure was performed for each of *n* sets, and the prediction error was computed using the index of the following formula:

$$\text{PRESS}=\sum_{i=1}^{n}{\left(Y_{i}-{\hat{Y}}_{i}\right)}^{2}$$

The number of components was selected in such a manner that the predicted residual sum of squares (*PRESS*) was minimized. In this study, statistical analysis was performed using EXCEL Multivariate Analysis Ver. 6.0 (Esumi Co., Ltd.).

## Results

### PTP1B inhibitory activity screening of 147 Kampo formulas

We screened the PTP1B inhibitory activity of all of the 147 oral Kampo formulations that are currently covered by health insurance in Japan. For a better approximation of its application in clinical settings, we compared the PTP1B inhibitory activity of each Kampo formulation based on the dose specified in the package inserts. We specifically defined the prescribed daily dose as 1 Unit (U) and 1/1,000 of this as 1 mU. Twenty-two of the 147 prescription Kampo formulations were demonstrated to completely inhibit the PTP1B activity (inhibition of >90%) at a final concentration of 0.1 mU/mL (Table 1).

**Table 1:** **The IC**_**50 **_**values of 22 Kampo prescriptions that demonstrated significant PTP1B inhibitory activity**

**Kampo formula**	**IC**_**50 **_**(μ Unit/mL)**^**a)**^
Daiokanzoto*	4.1 (± 0.08)
Masiningan*	4.3 (± 0.05)
Tokakujokito	4.9 (± 0.05)
Keimakakuhanto	6.3 (± 0.03)
Choijokito*	6.6 (± 0.09)
Bofutsushosan*	8.9 (± 0.08)
Daisaikoto	9.4 (± 0.06)
Kumibinroto	9.7 (± 0.63)
Kakkonto	9.7 (± 0.09)
Keishikashakuyakudaioto	10.2 (± 0.23)
Daijokito*	10.7(± 0.04)
Eppikajutsuto	11.6 (± 0.20)
Jidabokuippo	13.2 (± 0.04)
Tsudosan	13.8 (± 0.18)
Daisaikotokyodaio	14.0 (± 0.46)
Maoto	15.7 (± 0.18)
Kakkonkajutsubuto	15.7 (± 0.23)
Intinkoto	17.0 (± 0.20)
Maobushisaishinto	19.2 (± 0.16)
Kakkontokasenkyusini	19.8 (± 0.52)
Junchoto*	25.7 (± 0.94)
Keishito	29.5 (± 0.34)
*Rhei Rhizoma*	0.7 (± 0.02)^c)^
*Cannabisi Fructus*	51.6 (± 2.87)^c)^
Ursolic acid^b)^	4.3 (± 0.1)^d)^

The PTPlB inhibitory activity was significantly showed by 22 of the 147 Prescription Kampo Preparations. 22 prescriptions which showed high inhibitory activity calculate IC_50_^a)^Values are presented as mean ± SD, *n* = 3, Unit means the weight of a given dose for one day. ^b)^Positive control. ^c)^IC_50_ in μg/mL. ^d)^IC_50_ in μM. *These are classified into “Jyokito” and the medicine of relation in Chinese medicine.

### Dose-dependent assay and IC_50_

**Figure 1:**
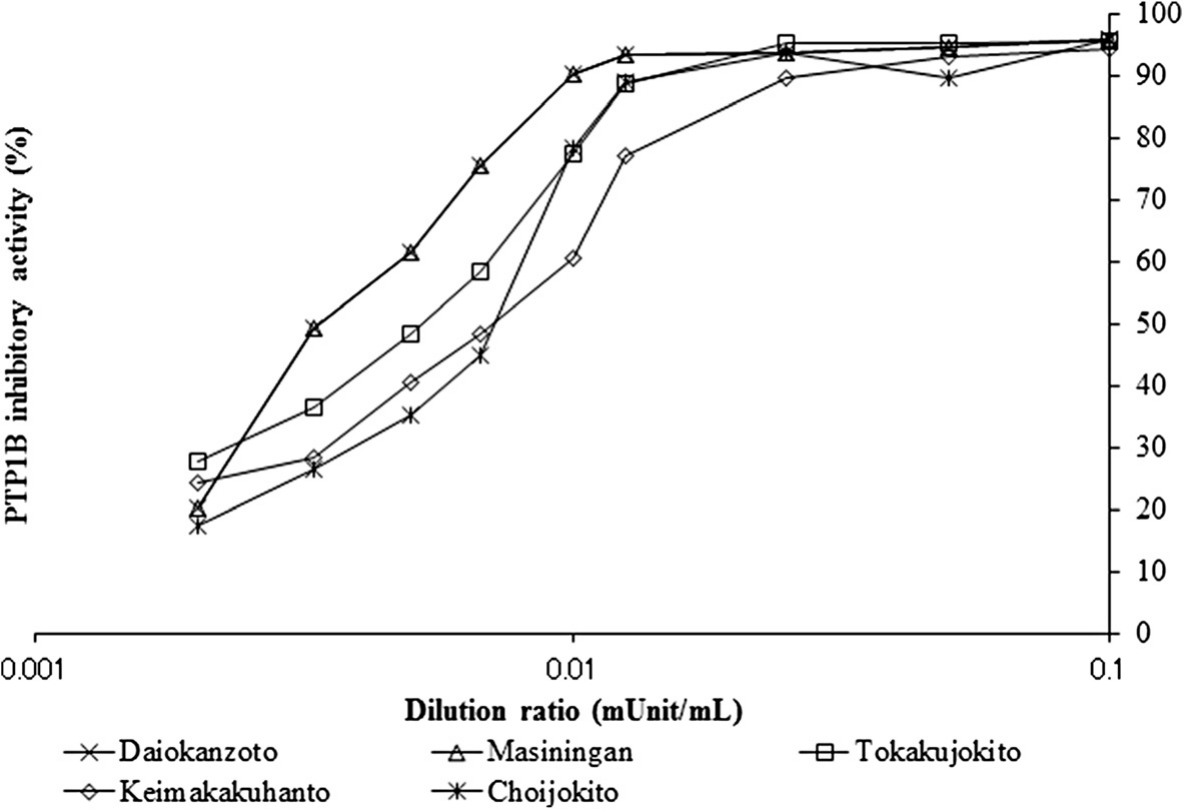
**Samples inhibit PTP1B in a dose-dependent manner.** Samples inhibit PTP1B in a dose-dependent manner. Different concentrations of the samples (0.002 – 0.1 mUnit/mL) were evaluated using the in vitro PTP1B enzyme assay. The dilution ratio is displayed on a logarithmic scale. The IC_50_ value was identified from the midpoint (cytotoxic activity = 50%) of the semi-log plot.

We investigated the dependence of the inhibitory activity on the concentration of 22 Kampo formulations that exhibited high PTP1B inhibitory activity. These 22 Kampo formulations were assayed for PTP1B inhibition at concentrations between 0.1 mU/mL and 0.001 mU/mL, and all displayed concentration-dependent inhibitory activity (Figure 1). To compare the inhibition potency of these Kampo formulations, their IC_50_ values were determined using the linear regression formula. Among the most potent was Daiokanzoto, followed by Masiningan, Tokakujokito, Keimakakuhanto and Choijokito.

### Kinetics analysis

**Figure 2:**
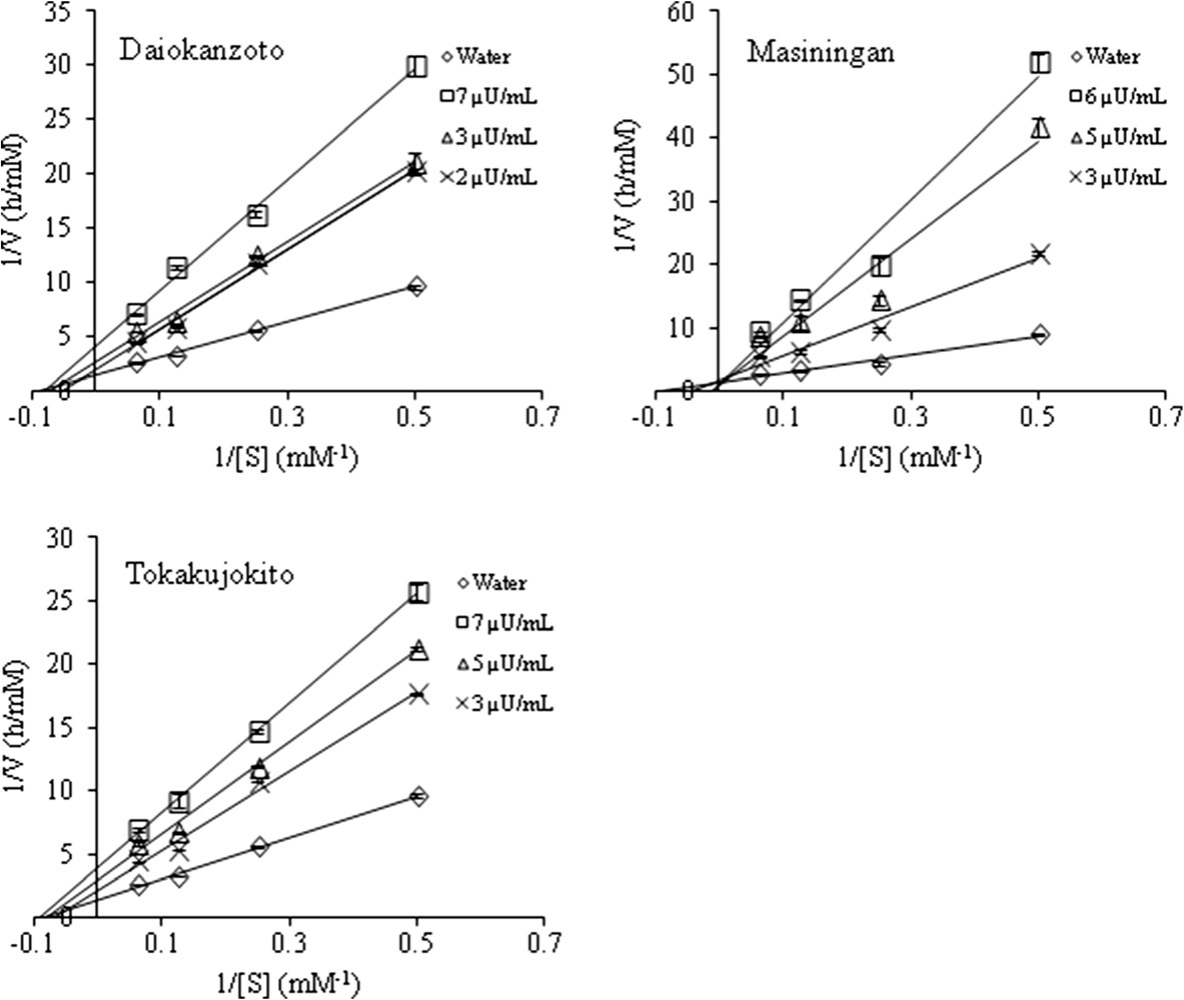
**Lineweaver–Burk Plots of the top three Kampo formulations.** Inhibition of PTP1B-catalyzed hydrolysis of *p*-NPP by Daiokanzoto, Masiningan and Tokakujokito. Lineweaver–Burk plot of the effect of the Kampo formulations on PTP1B. The final concentrations of Daiokanzoto were as follows: 0, 2, 3 and 7 μUnit/mL. The final concentrations of Masiningan were as follows: 0, 3, 5 and 6 μUnit/mL. The final concentrations of Tokakujokito were as follows: 0, 3, 5 and 7 μUnit/mL.

Daiokanzoto, Masiningan and Tokakujokito, which showed more potent inhibitory activity, were selected for further evaluation. Their inhibition mechanisms were elucidated using kinetics analysis with various concentrations of samples and the substrate, *p*-NPP. As shown in Figure 2, Lineweaver-Burk plots indicated that they inhibited PTP1B activity using mixed inhibition modes. However, the inhibition modes of Daiokanzoto and Tokakujokito were more non-competitive-like and Masiningan was competitive-like.

### Inhibitory selectivity

**Figure 3:**
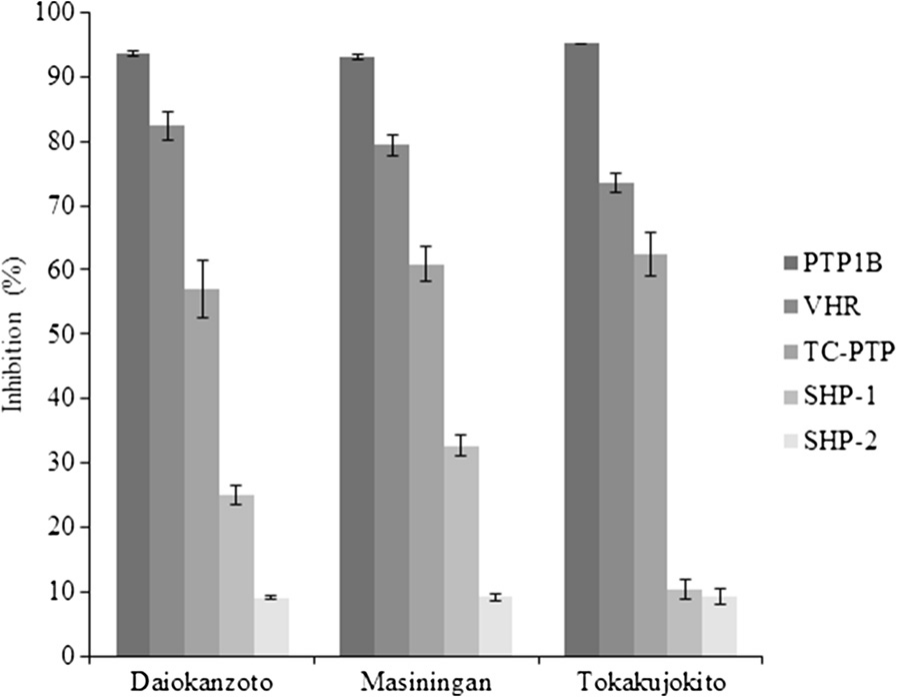
**Selectivity of the top three Kampo formulations against PTP1B, TCPTP, VHR, SHP-1 and SHP-2 at 25 μUnit/mL.** Inhibition selectivity of Daiokanzoto, Masiningan and Tokakujokito, which shows high PTP1B inhibitory activity against PTP1B, TCPTP, VHR, SHP-1 and SHP-2 at 25 μUnit/mL.

Due to the high structural similarity of the catalytic center among the family of protein tyrosine phosphatases [20], the inhibitory selectivity of Daiokanzoto, Masiningan and Tokakujokito were evaluated by comparing their inhibitory activity against PTP1B and four homologous protein tyrosine phosphatases: TCPTP, VHR, SHP-1 and SHP-2. At a final concentration of 25 μUnit/mL, these samples fully inhibited PTP1B activity, but partially inhibited other PTPs with different inhibition rate values: 73.6 – 82.4% against VHR, 57.0 – 62.4% against TC-PTP, 10.3 – 32.7% against SHP-1 and 9.0 – 9.2% for SHP-2 (Figure 3).

### Cytotoxicity and Akt phosphorylation assay

**Figure 4:**
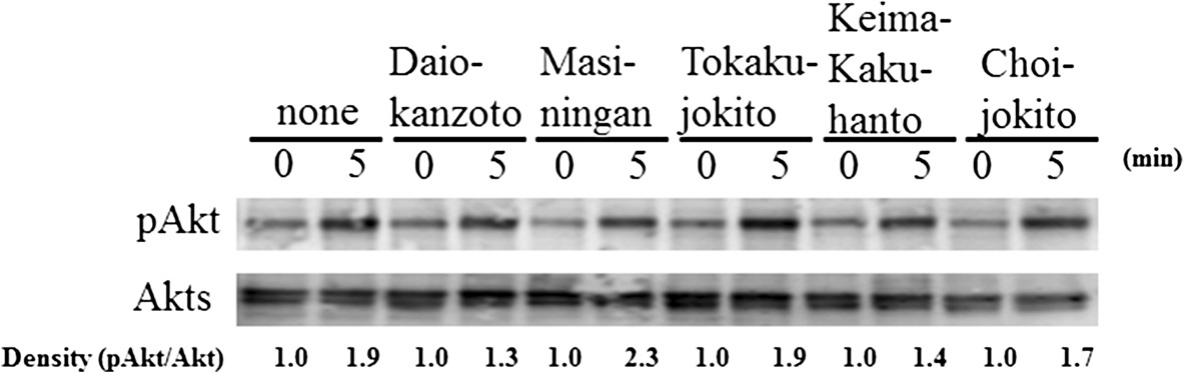
**Cellular activity of the Kampo formulations on Akt phosphorylation in HepG2 cells.** Cellular activity of the Kampo samples, which shows high PTP1B inhibitory activity, and water (negative control) on Akt phosphorylation in HepG2 cells.

On the basis of the enzymatic inhibition results, Daiokanzoto, Masiningan, Tokakujokito, Keimakakuhanto and Choijokito were further evaluated for their cellular activity in the insulin-signal-transduction pathway in the human hepatocellular carcinoma cell line, HepG2, by measuring the phosphorylation level of Akt, a key downstream effector of the insulin-signaling cascade. Because none of the samples showed cytotoxicity at a final concentration of 50 mU/mL in the Akt phosphorylation assay, the same sample concentration was used to assess their cellular activity in the insulin-signal-transduction pathway. After the cells were stimulated with insulin, the pAkt levels were analyzed using western blotting at different times (0, 5 min), in which water was used as a negative control. As shown in Figure 4, the rapid and transient increase in pAkt levels after insulin stimulation was promoted by the administration of Masiningan, but not by the other four samples.

### Partial least squares regression (PLS) analyses

The Kampo formulations primarily exerted their activities via the combination of multiple crude drugs. A statistical approach was used to identify the crude drugs with the greatest contribution to the PTP1B inhibitory activity of Kampo formulations that exhibited high PTP1B inhibitory activity. Given the large number of crude drugs constituting Kampo formulations and the colinearity observed for high correlations between these crude drugs, the five most potent Kampo formulations were selected for PLS analysis to investigate the contribution of the constituent crude drugs to PTP1B inhibition. We determined the optimum latent variable using the cross-validation model, and the coefficient of determination (R^2^) of the regression model was 0.947. The experimental value, predictive value and difference for each Kampo formulation are shown in Table 2. On the basis of the coefficients obtained for individual crude drugs in the regression model, *Rhei Rhizoma* exhibited the greatest contribution to PTP1B inhibitory activity among the 11 constituent crude drugs (Table 3).

**Table 2:** Predictive values of PTP1B inhibitory activity for the top five Kampo formulations used to construct the PLS model

**Kampo formula**	**Experimental value**	**Predictive value**	**Difference**^**a)**^
Daiokanzoto	4.1	4.4	−0.3
Masiningan	4.3	3.9	0.4
Tokakujokito	4.9	5.1	−0.2
Keimakakuhanto	6.3	6.3	0.0
Choijokito	6.6	6.4	0.2

Experimental value, Predictive value and Difference of each Kampo formula in the latent variable obtained by the cross- validation method are shown. The coefficient of determination in that case was 0.947. All values are expressed in μUnit/mL. ^a)^Difference = predicted - experimental.

**Table 3:** The regression coefficient of each constituent crude drug obtained using the PLS method

**Crude drug**	**Coefficient (a)**
*Rhei Rhizoma*	−0.017
*Cannabisi Fructus*	−0.010
*Armeniacae Semen*	−0.005
*Paeoniae Radix*	−0.005
*Cinnamomi Cortex*	−0.004
*Aurantii Fructus Immaturus*	−0.004
*Magnoliae Cortex*	−0.004
*Persicae Semen*	−0.003
*Zingiberis Rhizoma*	−0.001
*Zizyphi Fructus*	−0.001
*Ephedrae Herba*	−0.001

The crude drugs with the regression coefficients of 0 > (a) are shown. The size of a coefficient is reflected in a degree of incidence.

### PTP1B inhibitory activity of crude drugs

To confirm the results obtained using the PLS method, two crude drugs, *Rhei Rhizoma* and *Cannabisi Fructus* were selected to determine their PTP1B inhibitory activity. In the PTP1B inhibitory activity assay, both lyophilized decoctions of the crude drugs inhibited PTP1B activity in a concentration-dependent manner, and their IC_50_ values were calculated to be 0.7 and 51.6 μg/mL, respectively (Table 1). Because *Rhei Rhizoma* showed much more potent PTP1B inhibitory activity than *Cannabisi Fructus*, it is consistent with the results obtained from the statistical analysis.

## Discussion

Screening of drugs already in clinical use represents an optimal strategy used to identify a PTP1B inhibitor that is effective as a therapeutic agent against IR-T2DM, although no such drug is currently available for clinical use. The Kampo formulations are among the most promising candidates and are expected to exert integrative therapeutic effects. In the present study, we identified several Kampo formulations with high PTP1B inhibitory activity and *Rhei Rhizoma* as a constituent crude drug with the greatest contribution to the inhibitory activity.

High PTP1B inhibitory activity was associated with all of the formulations classified in Kampo medicine as Jyokito and its related formulations, including Daiokanzoto, Masiningan and Tokakujokito (Table 1). Jyokito formulations represent the most important group of prescription Kampo formulations to ameliorate “Ki” symptoms. One such formulation, Bofutsushosan, is already known to be effective for the treatment of metabolic syndromes, such as IR-T2DM and obesity [21]. The results from this study revealed the PTP1B inhibitory effect as an unforeseen feature shared by traditional Jyokito formulations.

The Kampo formulations, which exhibited the highest PTP1B inhibitory activity may be beneficial for diabetes mellitus treatment, even within the current limited scope of clinical application. For example, Daiokanzoto, Masiningan and Tokakujokito are all effective for the treatment of constipation, which is the most frequent gastrointestinal symptom observed in diabetic patients [22]. This represents a marked advantage in producing the integrative therapeutic effects expected in the clinical use of Kampo formulations.

Using the PLS method in multivariate analysis, *Rhei Rhizoma* exhibited the greatest contribution to the PTP1B inhibitory activity of Kampo formulations (Table 3), which was further supported by the extract of *Rhei Rhizoma* demonstrating more potent PTP1B inhibitory activity compared to *Cannabisi Fructus*. The significant effect of *Rhei Rhizoma* was demonstrated by comparing the PTP1B inhibitory activity of Kampo formulations containing highly related constituent crude drugs. Keishikashakuyakudaioto (IC_50_, 10.2 μUnit/mL) and Daisaikoto (IC_50_, 9.4 μUnit/mL) specifically exhibited higher inhibitory activity compared to Keishikashakuyakukanzoto (IC_50_, inactive) and Daisaikotokyodaio (IC_50_, 14.0 μUnit/mL). In addition to our present study, other studies have reported that *Rhei Rhizoma* is a beneficial crude drug for the amelioration of metabolic diseases from various perspectives, including diabetic nephropathy, hypercholesterolemia and vascular disorders [23,24]. Identification of the active substance in *Rhei Rhizoma* may result in the discovery of new therapeutic agents for IR-T2DM. Such a finding could further expand the potential of IR-T2DM treatment. Furthermore, further understanding of how the Kampo drug functions may be clarified by investigating reformulations of the complex PTP1B-inhibiting crude drug compositions in Kampo formulations.

## Conclusions

In conclusion, we successfully identified Kampo formulations with high PTP1B inhibitory activity from 147 prescription Kampo formulations. Although PTP1B is an important target for the treatment of diabetes mellitus, Kampo formulations exert their therapeutic effects via multiple mechanisms of action. Nevertheless, our study demonstrated the potential of concurrent assessments of Kampo formulations for an action on a single target for the purpose of identifying a candidate Kampo drug effective for the treatment of target-related diseases based on relevant theories in Kampo medicine, current knowledge in modern medicine and relationships found among constituent crude drugs from various perspectives. This may represent an innovative step toward the redefinition of the proper use of Kampo and the accumulation of evidence. On the basis of the results of this study, we are currently investigating how these candidate Kampo formulations, which were identified in this study, affect patients with IR-T2DM.

## Competing interests

The authors declare that there is no competing interests.

## Authors’ contributions

WL and KK conceived the study. WL, TO and KK designed this study. TO performed the experiments, and HK performed the cellular experiments. TO and WL performed data analyses and drafted the manuscript. All of the authors have read and approved the final manuscript.

## Pre-publication history

The pre-publication history for this paper can be accessed here:

http://www.biomedcentral.com/1472-6882/14/64/prepub
